# COVID-19, COPD, and AECOPD: Immunological, Epidemiological, and Clinical Aspects

**DOI:** 10.3389/fmed.2020.627278

**Published:** 2021-01-18

**Authors:** Francesca Polverino, Farrah Kheradmand

**Affiliations:** ^1^Asthma and Airway Disease Research Center, University of Arizona, Tucson, AZ, United States; ^2^Baylor College of Medicine, Houston, TX, United States

**Keywords:** COPD, COVID-19, SARS-CoV-2, cigarette smoke (CS), therapy, endothelium

## Abstract

The newly identified severe acute respiratory syndrome Coronavirus 2 (SARS-CoV-2) causes several heterogeneous clinical conditions collectively known as Coronavirus disease-19 (COVID-19). Older patients with significant cardiovascular conditions and chronic obstructive pulmonary disease (COPD) are predisposed to a more severe disease complicated with acute respiratory distress syndrome (ARDS), which is associated with high morbidity and mortality. COPD is associated with increased susceptibility to respiratory infections, and viruses are among the top causes of acute exacerbations of COPD (AECOPD). Thus, COVID-19 could represent the ultimate cause of AECOPD. This review will examine the pathobiological processes underlying SARS-CoV-2 infection, including the effects of cigarette smoke and COPD on the immune system and vascular endothelium, and the known effects of cigarette smoke on the onset and progression of COVID-19. We will also review the epidemiological data on COVID-19 prevalence and outcome in patients with COPD and analyze the pathobiological and clinical features of SARS-CoV-2 infection in the context of other known viral causes of AECOPD. Overall, SARS-CoV-2 shares common pathobiological and clinical features with other viral agents responsible for increased morbidity, thus representing a novel cause of AECOPD with the potential for a more long-term adverse impact. Longitudinal studies aimed at COPD patients surviving COVID-19 are needed to identify therapeutic targets for SARS-CoV2 and prevent the disease's burden in this vulnerable population.

## Introduction

*COVID-19*: Coronavirus disease-19 (COVID-19) is an acute and heterogeneous clinical condition caused by the newly identified severe acute respiratory syndrome Coronavirus 2 (SARS-CoV-2). In most cases, the disease is characterized by a mild presentation of respiratory symptoms and/or other extra-pulmonary conditions such as anosmia, gastrointestinal distress, and malaise. When severe, the disease can lead to acute lung injury (ALI) and acute respiratory distress syndrome (ARDS) which carry high morbidity and mortality. Despite the tremendous worldwide burden, our understanding of the pathophysiology and treatment of SARS-CoV-2 illnesses remains limited.

The elderly and those with comorbidities (mainly cardio/respiratory diseases, diabetes, and kidney failure) carry the highest risk of poor prognosis and death (1–5). Chronic Obstructive Pulmonary Disease (COPD) is one of the most frequent chronic respiratory conditions affecting millions worldwide, and is mainly caused by exposure to cigarette smoke. COPD predominantly affects smokers in their sixth or seventh decade of life, and leads to deconditioning with frequent comorbid conditions. Because of off-target immune responses (6), COPD is associated with increased susceptibility to infections, including respiratory viral diseases that are among the leading causes of acute exacerbations of COPD (AECOPD) (7). Therefore, predictably SARS-CoV2 pandemic has critically impacted COPD patients (8).

Here, we discuss the epidemiology of COVID-19 in the COPD population, the clinical and therapeutic implications of COVID-19 as the ultimate cause of AECOPD, and the pathological and immunological factors that play key roles in ever smokers with and without COPD affecting SARS-CoV2 infection and COVID-19 outcome.

## COVID-19 and COPD: Epidemiology

The majority of COPD patients are older, and have frequent comorbid conditions, making those diagnosed with COVID-19 display with worse outcomes, including a higher incidence of hospitalization, Intensive Care Unit (ICU) admission, and mortality (9, 10). However, it remains to be determined how pre-existing chronic inflammatory airway diseases, such as COPD, and their treatment might modify the risk for SARS-CoV-2 infection and development of COVID-19.

In mainland China and worldwide, several cohort studies showed a lower prevalence of active smokers than expected gender and age-adjusted prevalence among COVID-19 patients (1–4, 11–13). Whether this is caused by incomplete or incorrect information about smoking patterns due to significant methodological limitations, including small sample sizes and ascertainment bias, remains unclear. Similarly, the data on COPD among COVID-19 patients revealed a wide-ranging prevalence (1.1–38%) of COPD among patients hospitalized with COVID-19. To estimate the COPD patient excess risk for contracting COVID-19 has been challenging since reporting has concentrated on hospitalized and ICU patients rather than on milder (e.g., outpatient settings) cases. Further, not all the COPD patients with COVID-19 sought medical treatment in a hospital setting because of overburdened hospitals at the peak of pandemic. Thus, COVID-19 related AECOPD may have contributed to the mortality statistics without having affected the hospitalization or ICU-related statistics (14). Understandably, pulmonary function tests are not performed in COVID-19 patients before or after hospitalization, and thus, airflow limitation might have been underestimated.

A meta-analysis of 15 studies where the presence of COPD was assessed among 2,473 confirmed COVID-19 patients revealed that the pooled prevalence of COPD diagnosis in COVID-19 patients was relatively low [2% [95% CI, 1%−3%]]. Whether this observed effect is due to self-isolation of COPD patients resulting in lower representations in some of the COVID cohorts, remains less clear. However, COPD patients were at a higher risk of more severe disease burden, and significantly higher risk of mortality, compared to controls (calculated RR, 1.88) (8). Another cohort study with covariate adjustment using multivariate logistic regression revealed no significant differences in the rate of SARS-CoV-2 positivity between COPD and non-COPD patients. However, significantly higher rates of hospitalization [adjusted odds ratio [OR]of 1.36], ICU admissions (adjusted OR of 1.20), and invasive mechanical ventilation (adjusted OR of 1.49) were observed in COPD patients infected with the virus when compared with non-COPD patients (15).

Taken together, if, on the one hand, published studies show a diverse prevalence of COPD among COVID-19 patients, on the other hand, they consistently show a worse clinical outcome in the same population. Further studies are needed to rule out potential ascertainment biases in profiling the COPD COVID-19 population and establishing whether the graver outcome in COPD patients is due to their underlying lung disease, the nature of the immune responses, the underlying pharmacologic COPD treatment (e.g., chronic steroid use), or a combination thereof.

## AECOPD and COVID-19

Coronaviruses are seasonal causes of AECOPD. Several factors can play a role in the increased susceptibility to develop severe SARS-CoV-2 infection in COPD patients. First, COPD patients often use many metered dose inhalant medications such as corticosteroids (ICS). The study by Attaway et al. (15) showed that COPD patients who tested positive for SARS-CoV-2 were 2.4 times less likely to have used corticosteroids at the time of testing than those who tested negative. Notwithstanding a few significant methodological limitations, such as proper control for confounding factors including treatment indication and disease severity, they raise a question about corticosteroids' role in COVID-19. ICS and long-acting bronchodilators are first-line therapies for AECOPD. When appropriate, AECOPD can be managed with antibiotics and oral corticosteroids (OCS). AECOPD directly related to SARS-CoV-2 infection should be treated with OCS such as dexamethasone, especially if they require supplemental oxygenation or invasive mechanical ventilation.

Further, in a randomized study of patients hospitalized with COVID-19, dexamethasone use resulted in lower 28-day mortality among those requiring either invasive mechanical ventilation or oxygen alone but not among those not requiring supplemental oxygen (16). On the other hand, long-term treatment with systemic corticosteroids is immunosuppressive, increasing the risk and severity of viral infections. Chronic impairment of the innate and acquired antiviral immune responses results in delayed viral clearance, enhanced mucus production, impaired antimicrobial peptide secretion, and increased pulmonary bacterial load during virus-induced exacerbations (17). Thus, the use of ICS during this pandemic has been called into question (18). Notably, a recent meta-analysis on COVID-19 outcomes in patients with chronic respiratory diseases using ICS concluded that there is currently no evidence on benefits or harms in COVID-19 in patients with underlying chronic respiratory illnesses (19). Consistently, an observational study using the OpenSafely platform that assessed more than 140,000 COPD patients, did not find a protective role for regular ICS use against COVID-19-related death in COPD patients (20). The Global Initiative for Obstructive Lung Disease (GOLD) recommendations recently released a statement confirming the lack of scientific evidence to support that ICS or OCS should be avoided in patients with COPD during the COVID-19 epidemic (www.goldcopd.org). Research efforts should focus on profiling the characteristics of COVID-19, who could have more benefit from the use of ICS than potential complications.

## COPD, ACE2, and SARS-CoV2: Matters of Debate

COPD emerges from a complex interaction between genetic predisposition and environmental factors, while cigarette smoke remains the leading environmental cause of COPD. Between 15% and 50% of smokers develop COPD, 80–90% of COPD patients are smokers or ex-smokers (21, 22). To date, no definitive evidence on whether ever-smokers are at an increased risk of SARS-CoV-2 infection exists.

**Figure 1:**
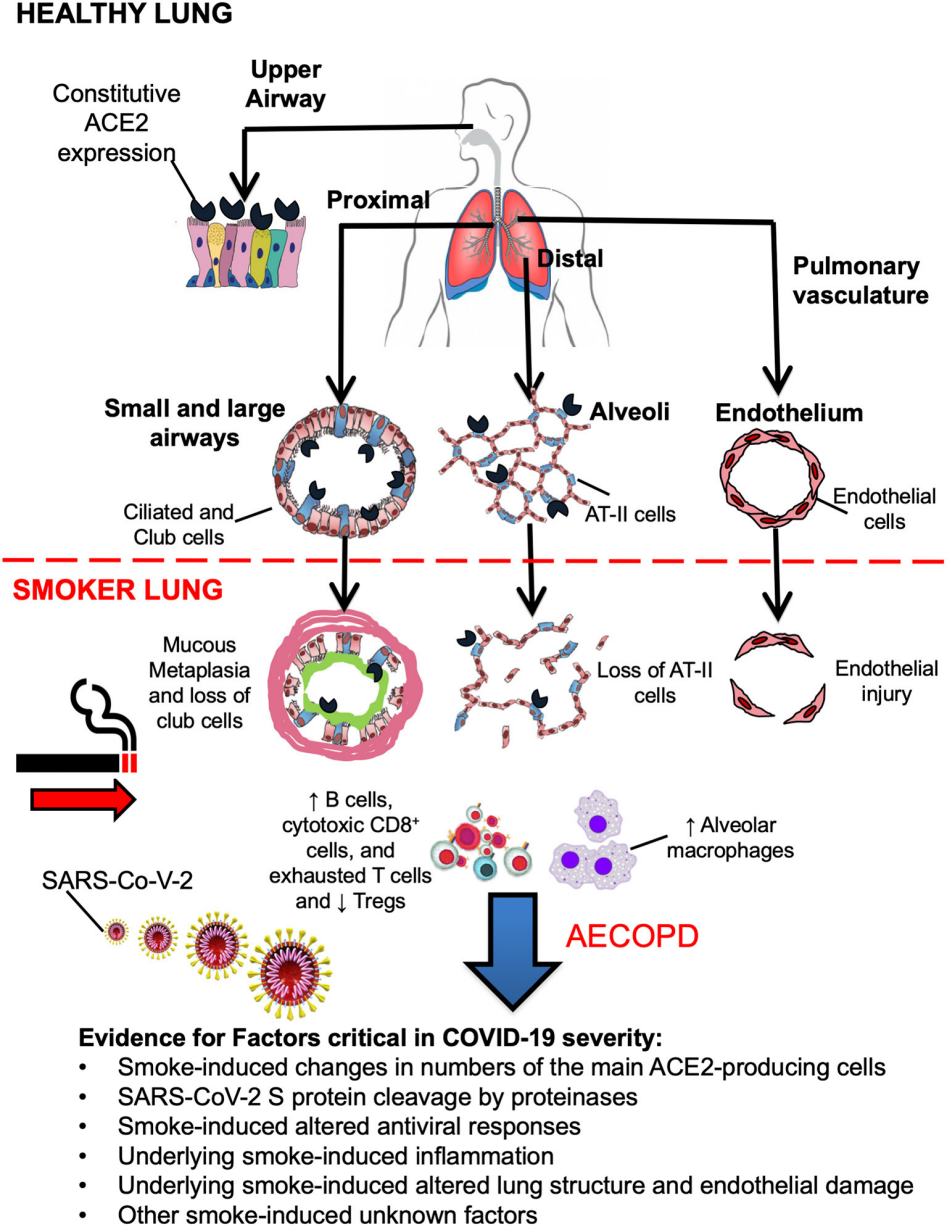
There is a gradient of ACE2 expression throughout the respiratory tract. The highest ACE2 expression is found in the nasal epithelium followed by the larger airway epithelium, waning in the more distal bronchiolar and alveolar lung regions, in particular ciliated and secretory club cells, and alveolar type II (AT-II) cells. Cigarette smoke induces a loss of club cells and AT-II cells and extensive hyperplasia of goblet cells, but whether this is associated with increased susceptibility to SARS-CoV-2 infection is unclear. Additionally, cigarette smoke induces increases in the numbers of alveolar macrophages, that are the main cells expressing proteinases, and upregulates B cells, cytotoxic CD8^+^ T cells often with an exhausted phenotype, while downregulating the T regulatory cell compartment. This chronic impairment of the innate and acquired immune responses results in delayed viral clearance, enhanced mucus production, impaired antimicrobial peptide secretion, and increased pulmonary bacterial load during virus-induced exacerbations and possibly during SARS-CoV-2 infection.

There are several possible ways cigarette smoke and COPD could affect SARS-CoV-2 entry and COVID-19 development (see Figure 1). SARS-CoV-2 uses the angiotensin-converting enzyme 2 (ACE2) as the host cell entry receptor (23). High-sensitivity RNA *in situ* mappings revealed the highest ACE2 expression in the nasal epithelium (24) with decreasing expression throughout the lower respiratory tract. Similarly, *in vitro* studies showed a striking gradient of SARS-CoV-2 infection in proximal (high) vs. distal (low) pulmonary epithelial cultures (24). Additionally, the expression of ACE2 in the lower respiratory tract and the upper respiratory tract is under different regulation (25). ACE2 gene in the peripheral lung has been found mainly expressed in goblet cells in smokers and club cells and alveolar type 2 (AT-II) cells in never smokers (23, 26).

Interestingly, cigarette smoke remodels cells in the bronchial epithelium inducing mucous cell metaplasia with a loss of club cells and apoptosis of alveolar septal cells (particularly AT-II cells) (27, 28), but this does not necessarily translate into higher SARS-CoV-2 infectivity. Notably, *in vitro* studies have shown that, whereas the ciliated cells are infected with SARS-CoV-2 and become extruded, the MUC5B+ club cells, or MUC5AC+ metaplastic goblet cells in the airway lumen, express high levels of ACE2 but are not easily infected (24). When conflated with features of attributable COVID-19 lung morbidity, these studies show quite persuasively that lung ACE2 expression is a suboptimal marker of SARS-CoV-2 infection and COVID-19 disease expression and morbidity.

Most of the studies published so far have implied that increases in lung ACE2 observed in ever smokers with and/or without COPD indicate greater susceptibility to SARS-CoV-2 infection and its expected complications (13, 26, 29, 30). However, there are several conflicting factors to consider when assessing the effects of cigarette smoke on ACE2 and SARS-CoV-2 infection. First, if, on the one hand, cigarette smoke and COPD have been associated with elevated pulmonary ACE2 levels (26), it is unclear whether this translates into higher SARS-CoV-2 infectivity and worse disease. A pivotal study published by Imai et al. (31) showed that ACE2 protects mice from severe ALI induced by acid aspiration or sepsis, and loss of ACE2 resulted in increased pulmonary inflammation and vascular permeability, causing worsening of the ARDS. Second, nicotine interacts with many components of the renin-angiotensin system (RAS) in multiple organ systems. In the ACE/ Angiotensin II (AT-II) / Angiotensin_1_ Receptor (AT_1_R) arm, nicotine increases the expression and/or activity of renin, ACE, and AT_1_R, whereas, in the compensatory ACE2/Angiotensin-(1–7) arm, nicotine downregulates the expression and/or activity of ACE2 and Angiotensin II Receptor (32–34). Third, cigarette smoke [both via activation of nicotinic acetylcholine receptors abundant in the bronchial epithelium (35), and via upregulation of macrophages] (36) can lead to enhanced activity of proteinases that can cleave SARS-CoV-2 S protein leading to viral entry into the host cells (37), and cause ACE2 shedding thus increasing viral uptake (38, 39). Fourth, soluble ACE2 can be released from the epithelial surface into the airways via cleavage by sheddases (38, 39). The release of soluble ACE2 is a dynamic process, occurring both constitutively and in response to various stimuli, possibly cigarette smoke and COPD. This process may activate off-targeted innate and adaptive immune responses and play a role in SARS-CoV-2 infection, as described below.

## The Immune System in COPD: Implications During SARS-CoV-2 Infection

COPD patients have blunted protective immune responses (6), affecting the mucociliary clearance and the activity of alveolar macrophages (40), dendritic cells (41), cytotoxic and regulatory T cells (42, 43), B cells and mucosal antibody production (44–46). Importantly, anti-viral immune responses such as Irf-7, IFN-α, factors contribute to the and IFN-β are significantly compromised in the lungs of active smokers (17, 47, 48). These factors contribute to the increased susceptibility to infections, and viral infections are one of the main causes of AECOPD.

Cigarette smoke compromises cellular anti-viral defenses via several mechanisms that involve both innate and adaptive immune responses. For example, exposure to cigarette smoke increases the numbers of alveolar macrophages in COPD. However, their phagocytic ability, anti-viral mediator expression, and clearance of apoptotic cells are reduced in COPD (36, 49–51). In particular, some of the macrophage-derived proteinases (e.g., A Disintegrin and A Metalloproteinase 17; ADAM17) can shed both SARS-CoV-2 S protein, inducing viral entry into the host cells (37), and ACE2 (38, 39) releasing it from the epithelial surface into the airways. Further, IFN-β, a critical cytokine required to inhibit coronavirus replication (52), is significantly reduced in lung epithelium and alveolar macrophages of COPD patients (47). Similarly, cigarette smoke stimulates specific serine phosphorylation-dependent ubiquitination and degradation of a type I IFN receptor subunit leading to attenuation of IFN signaling and decreased resistance to viral infection (53).

Cigarette smoke exposure also affects the adaptive immune system by inducing off-targeted T and B cell responses (42, 54, 55) that could contribute to worsening of SARS-CoV-2 infection and clinical outcome. B cells are critical players in the mucosal adaptive immune responses, leading to soluble IgA (sIgA) production against microbial and/or self-antigens (56, 57). There is a downregulation of the epithelial polymeric immunoglobulin receptor (pIgR, essential for the generation of mucosal sIgA) (58), thus causing an impaired viral clearance in COPD. Further in the emphysematous phenotype, there is an abundance of memory B cells that is not common in the lungs of subjects without COPD (54). The T cell compartment is also affected by the presence of COPD. T cells in the COPD lung tend to express an exhausted phenotype with reduced cytotoxicity and upregulation of programmed cell death protein-1 (PD-1) expression, which renders immunosuppressive actions on CD8^+^ cells (59), paralleled by an expansion of oligoclonal CD4^+^ T cells in lung tissue from COPD patients (60). Additionally, COPD patients have decreased numbers of circulating and pulmonary Treg cells, and reduced levels of FoxP3 mRNA and lung interleukin 10 (IL-10) secretion compared with never and ever smokers without COPD (42, 46).

The pathogenesis of COVID-19 has some immune features in common with COPD. CD4 T and CD8 T cell numbers are substantially decreased, whereas the Th17 and CD8 cytotoxic T cell populations are significantly grown in the peripheral blood from COVID-19 patients with ARDS (61). Instead, the SARS-CoV-2 infected pulmonary tissue's main changes include increases in natural killer (NK) cell, macrophage (mainly type I macrophage), B cell, and dendritic cell numbers, which contribute to the activation of CD8 and CD4 T cells (61). Simultaneously, the presence of exhausted T cells and a decrease in a blunted T regulatory compartment has been described in both blood and lung from COVID-19 patients (62, 63), likely contributing to the inflammatory storm and the pulmonary tissue damage observed in severe COVID-19. Also, a shift of the B cell compartment toward “atypical” memory B cells (lacking CD27 and low CD21 expression, unlike classical B cells that are CD27^+^ CD21^+^), described in other viral infections such as HIV, HBV, or HCV, have been described in COVID-19 patients (64), but the exact role of these atypical B cells needs to be clarified.

Overall, while it is unclear whether ever smokers with and/or without COPD may be at higher risk of SARS-CoV-2-infection (e.g., due to cigarette smoke -induced changes in numbers of the main ACE2-producing cells in the lung and/or increased SARS-CoV-2 S protein cleavage) if infected, they may be more severe due to underlying pulmonary inflammation and altered lung structure due to remodeling. Additional studies, both targeting cigarette smoke -exposed animals and *in vitro* models, are needed to shed light on the mechanisms by which cigarette smoke and COPD affect SARS-CoV-2 entry in the host cells and cause pulmonary disease.

## Endothelial Dysfunction: A Common Culprit in COPD and COVID-19

In addition to the lungs, chronic cigarette smoke has been linked to dysfunction in multiple organs including the kidney, and systemic vascular endothelial dysfunction or injury (65, 66). Cigarette smoke-induced endothelial dysfunction can manifest as arterial hypertension (~70% of COPD patients), atherosclerosis, systemic inflammation, pulmonary arterial hypertension, cor-pulmonale, venous thromboembolism, and microalbuminuria (67).

A number of mechanisms underlie endothelial injury/dysfunction in COPD which include: (1) direct toxic effects of cigarette smoke on endothelial cells; (2) generation of auto-antibodies directed against endothelial cells; (3) vascular inflammation and oxidative stress, with reduced activation of the anti-oxidant pathways in endothelial cells; (4) increase in endothelial cell release of mediators with vasoconstrictor, pro-inflammatory, and remodeling activities (endothelin-1) and reduced endothelial cell expression of mediators that promote vasodilation and homeostasis of endothelial cells, such as nitric oxide and prostacyclin; 5) increased endoplasmic reticular stress and the unfolded protein response in endothelial cells; and (6) decreased expression of Vascular Endothelial Growth Factor (VEGF) associated with reduced hypoxia inducible factor- 1, a transcription factor that drives the expression of genes involved in endothelial function (66, 68).

Notably, some of the same pathological mechanisms in cigarette smoke-mediated endothelial dysfunction are hallmarks of respiratory viral infections, including SARS-CoV-2 infection. Clinically, coronavirus-infected patients show hypercoagulable state with thrombosis, with fibrin clots being one of the most common histopathological findings in the lungs and other organs from COVID-19 patients (69).

SARS-CoV-2 infection triggers an inflammatory cascade with increased IL-6, C-reactive protein, erythrocyte sedimentation rate, and elevated fibrinogen at presentation (70). Some patients appear to have a more pronounced inflammatory response to infection with SARS-CoV-2, such as seen with systemic inflammatory response syndrome or cytokine storm. Importantly, increased IL-6 levels correlate with increased fibrinogen, further supportin a strong link between inflammation and procoagulant changes (71). Together, the pulmonary and systemic effects of cigarette smoke could further potentiate SARS-CoV-2-induced endothelial dysfunction and the systemic inflammation in COPD patients. The mechanisms underlying this potential synergy, remains elusive. Nicotine interacts with many components of the renin-angiotensin system in multiple organ systems by decreasing ACE2 levels, thus indirectly regulating the vascular tone (34), but it is unknown whether cigarette smoke and/or COPD directly decrease ACE2 expression in the endothelial cells. Similarly, it is unclear whether cigarette smoke and/or COPD exacerbate inflammation in COVID-19 patients, since previous studies point at both inflammatory and anti-inflammatory effects of cigarette smoke (72). Further studies are needed in order to elucidate the direct effects of cigarette smoke and COPD on endothelial cells in the context of SARS-CoV-2 infection.

## Conclusions

Overall, the effect of cigarette smoke on initial sites of SARS-CoV-2 infection, the mechanisms that seed infection distally in the lungs, and the virus-host interactions that attenuate or augment intra-regional virus growth in the cigarette smoke -exposed lung to produce severe disease are unclear (Figure 1). We suggest that *in vivo* and *in vitro* models aimed at testing the effects of cigarette smoke on SARS-CoV-2 infection could shed light needed insight. Some of the unmet needs and knowledge gaps identified as follows:

- How does SARS-CoV-2 infection affect different types of COPD endotypes (e.g., emphysema-predominant, chronic bronchitis, eosinophilic-predominant, frequent exacerbators)?; what is/are the long-term effect/s of COVID-19 on their systemic and respiratory outcome?
- Should the gold standard recommendations for treating COPD and AECOPD (bronchodilators + corticosteroids) be applicable in COVID-19? Or should they be personalized according to the underlying COPD phenotype?
- How broadly should we expand the current therapeutic, radiological, and clinical follow-up of COPD patients post-COVID-19 infection to better understand its impact on disease progression?
- Are ever smokers with and/or without COPD at increased risk of getting infected with SARS-CoV-2? Moreover, if infected, are they at increased risk of developing severe COVID-19 outcomes?
- How do the innate and adaptive immune systems, and the anatomic changes associated with cigarette smoke exposure that play a significant role in the pathogenesis of COPD, affect the COVID-19 outcome in ever smokers with and/or without COPD?
- How does cigarette smoke and COPD affect the endothelium in COVID-19 patients? Do they exacerbate the endothelial dysfunction known to be key in the pathogenesis of COVID-19?

## Author Contributions

All authors listed have made a substantial, direct and intellectual contribution to the work, and approved it for publication.

## Conflict of Interest

The authors declare that the research was conducted in the absence of any commercial or financial relationships that could be construed as a potential conflict of interest. The handling Editor declared a past co-authorship with one of the authors FP.
